# Two-Photon Microscopy Analysis of Gold Nanoparticle Uptake in 3D Cell Spheroids

**DOI:** 10.1371/journal.pone.0167548

**Published:** 2016-12-09

**Authors:** Tushar D. Rane, Andrea M. Armani

**Affiliations:** Mork Family Department of Chemical Engineering & Materials Science, University of Southern California, Los Angeles, California, United States of America; Universidade Nova de Lisboa, PORTUGAL

## Abstract

Nanomaterials can be synthesized from a wide range of material systems in numerous morphologies, creating an extremely diverse portfolio. As result of this tunability, these materials are emerging as a new class of nanotherapeutics and imaging agents. One particularly interesting nanomaterial is the gold nanoparticle. Due to its inherent biocompatibility and tunable photothermal behavior, it has made a rapid transition from the lab setting to *in vivo* testing. In most nanotherapeutic applications, the efficacy of the agent is directly related to the target of interest. However, the optimization of the AuNP size and shape for efficacy *in vitro*, prior to testing in *in vivo* models of a disease, has been largely limited to two dimensional monolayers of cells. Two dimensional cell cultures are unable to reproduce conditions experienced by AuNP in the body. In this article, we systematically investigate the effect of different properties of AuNP on the penetration depth into 3D cell spheroids using two-photon microscopy. The 3D spheroids are formed from the HCT116 cell line, a colorectal carcinoma cell line. In addition to studying different sizes and shapes of AuNPs, we also study the effect of an oligo surface chemistry. There is a significant difference between AuNP uptake profiles in the 2D monolayers of cells as compared to the 3D cell spheroids. Additionally, the range of sizes and shapes studied here also exhibit marked differences in uptake penetration depth and efficacy. Finally, our results demonstrate that two-photon microscopy enables quantitative AuNP localization and concentration data to be obtained at the single spheroid level without fluorescent labeling of the AuNP, thus, providing a viable technique for large scale screening of AuNP properties in 3D cell spheroids as compared to tedious and time consuming techniques like electron microscopy.

## Introduction

Nanomaterials hold tremendous promise for targeted delivery of next generation cancer therapies[1–3]. These materials can play numerous roles in an overall nanotherapeutic design strategy, from acting as a protective encapsulant to playing the role of the actual therapeutic agent. One unique class of nanotherapeutics is the gold nanoparticle (AuNPs). AuNPs have been used as drug carriers[4], photothermal agents[5], contrast agents[6] and radiosensitizers[7]. Their broad impact can be directly related to the ease of synthesis, shape control, tunable surface functionalities, and biocompatibility[8].

A critical factor involved in the efficacy of AuNPs as therapeutic agents is their ability to penetrate the target of interest (e.g. tumors in cancer). While systematically varying all parameters of the AuNP, such as size, shape and surface functionalization, *in vivo* would be ideal, these experiments are extremely expensive, time consuming, and ill-suited for large scale testing. As a compromise, AuNP uptake is typically screened in a 2D monolayer of cells for several AuNP variations, prior to shortlisting a few variations for *in vivo* testing. It has been well established, however, that behavior of cells in 2D monolayers can be very different from behavior of cells in 3D tissue [9–11]. In cancer, for instance, drug candidates found to be effective in 2D monolayers are less effective in 3D on one hand while some potential targets for therapy exclusively play a role in 3D environment[12].

**Fig 1:**
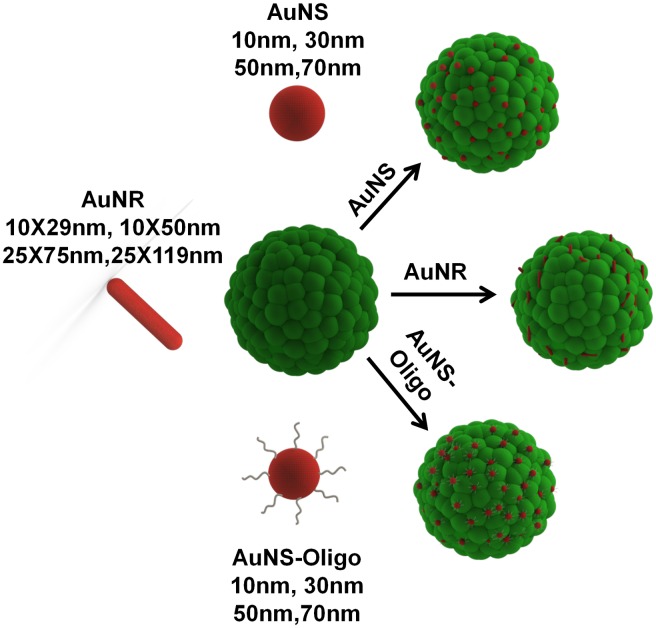
Schematic indicating series of gold nanoparticle incubation experiments with cell spheroids to determine optimal nanoparticle size, shape and surface chemistry for efficient therapeutic delivery.

In the present work, we perform a comprehensive study using a suite of gold nanoparticles and comparing the results from 2D cultures with 3D spheroids. By systematically varying the nanoparticle size, shape, and surface chemistry as well as the cell culture strategy, we determine the optimum nanoparticle geometry to maximize delivery to the spheroid. An overview of the multi-faceted series of measurements is contained in Fig 1.

## Results and Discussion

The 2D cell cultures and the 3D cell spheroids were generated using a TurboGFP expressing HCT116 cell line, a colorectal carcinoma cell line. We established conditions suitable to grow cell spheroids approximately 400μm in diameter. This diameter was selected based on the field of view of the two-photon microscope. 2D cell cultures for imaging were generated in standard cell culture plates by seeding HCT116 cells at densities which resulted in approximately 80% confluency after 3 days of culture.

As specified in Fig 1, the key variables studied were size, shape, and surface chemistry. Four different diameter spherical gold nanoparticles (AuNS) and four different gold nanorods (AuNR) with two different small diameters and two different aspect ratios were used. The diameters and aspect ratios of AuNPs tested are indicated in the Methods section while the characterization of the AuNPs using UV-Vis spectrophotometry is included in the supplement (Fig A in S1 File). In addition, surface functionalization is an important parameter that can be modified to engineer the uptake levels of AuNP in cells, and oligonucleotides form one class of molecules that have been used previously to functionalize AuNP surfaces for enhanced uptake[13,14]. To test the effects of AuNP surface functionalization, we also functionalized the AuNS with 28base long oligonucleotides (AuNS-oligos). The oligonucleotides were functionalized with dithiol on the 5’ end for conjugation to the gold surface and Alexa594 dye on the 3’ end to enable verification of the conjugation using fluorescence spectroscopy (Fig B in S1 File). To determine the impact of the nanomaterials on the cell cultures, we focused on three metrics: growth rate, uptake efficiency, and penetration depth into the spheroid.

**Fig 2:**
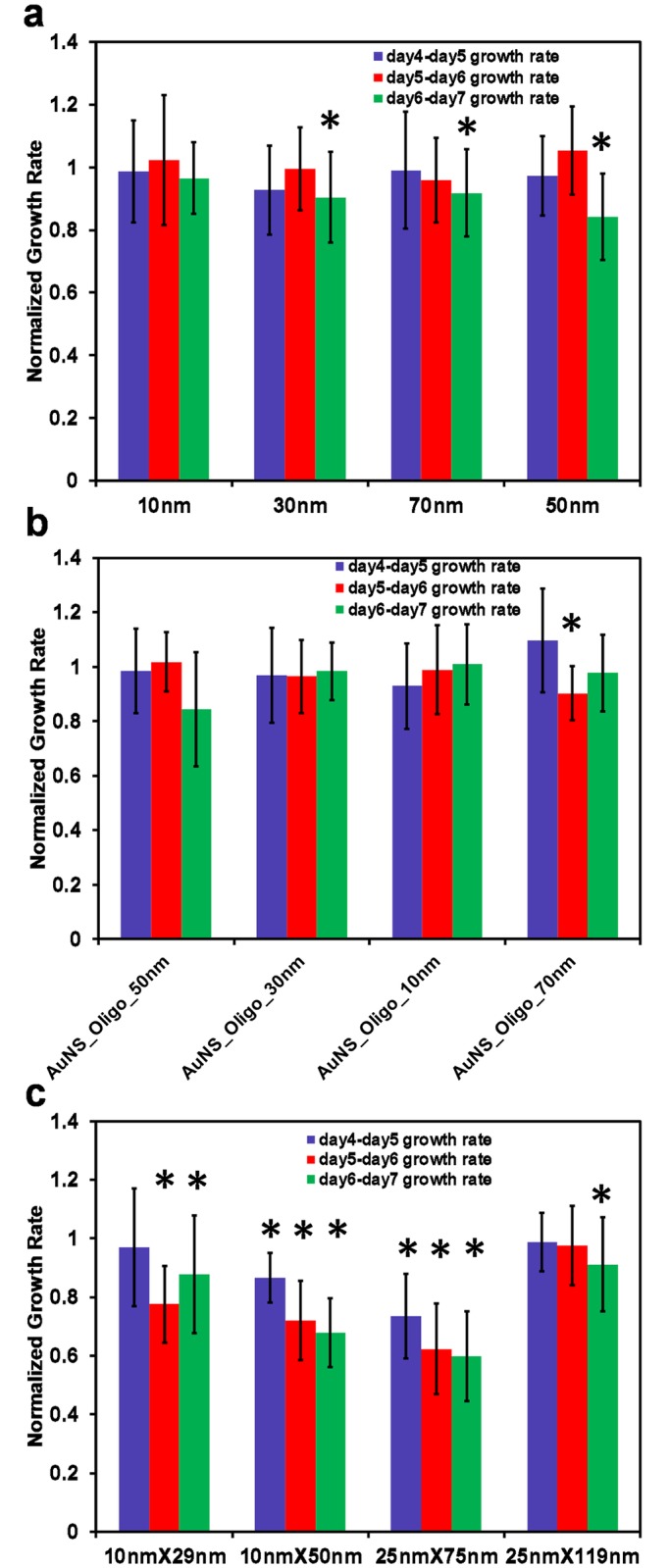
Growth rate of cell spheroids exposed to the different AuNPs for 72 hours, normalized to the growth rate of control spheroids cultured without AuNP. a)AuNS, b) AuNS-oligos, and c)AuNR. The number of spheroids used for calculating the growth rate for each condition ranged from 5 to 8. The ‘*’s in the figure indicates a positive T-test testing the hypothesis that the normalized growth rate is less than 1 at a significance level of 0.001.

The growth rate of the spheroids exposed to various AuNP normalized to the growth rate of control spheroids as determined by phase contrast microscopy is plotted in Fig 2 (details included the Methods section). If the AuNPs in the media have no effect on incubation, the growth rate normalized with control spheroid growth rate would be approximately 1 while growth rate reduction by AuNP will result in the normalized growth rate being significantly less than 1.

For the concentration and size range of AuNPs we tested, the AuNS appear to have minimal effect on the growth rate of the 3D spheroids (Fig 2a and 2b). Similarly, incubation of 2D cultures with AuNS qualitatively appears to have no effect on the growth rate of the 2D cultures (Fig C in S1 File). In contrast, AuNR incubation had a significant effect on the 2D and 3D cell cultures. The majority of the cells died in the 2D cell cultures when exposed to AuNR (Fig C in S1 File). When the spheroids were exposed to identical AuNR concentrations in 3D, only two of the AuNR geometries had a minimal impact on the growth rate. This decrease is most likely the result of the cytotoxic CTAB stabilizing agent in the AuNR solution[15–17]. This result further strengthens the hypothesis that the results obtained from 2D cultures may not be representative of the *in vivo* behavior of a therapeutic agent.

To accurately quantify the uptake efficiency in the 3D spheroid, it is necessary to measure both the nanoparticle concentration in the spheroid and nanoparticle location. In past work, ICP-MS has been the technique of choice to measure uptake of AuNP in cells[18,19] due to the very high sensitivity of the technique. However, this method is unable to determine location within a sample. To overcome this problem, ICP-MS results are frequently complemented with TEM imaging, in which cross sections of a sample are taken. TEM imaging, however, is extremely tedious and time consuming, especially to screen a large number of nanoparticle types with replicates, if quantitative measurements are desired.

An alternative imaging strategy is two-photon photoluminescence (TPPL) microscopy. Similar to confocal microscopy, this non-destructive imaging technique allows for monitoring of the particle location within the spheroid. Because gold nanoparticles exhibit TPPL, there is no need for a secondary labeling molecule[20]. Two-photon microscopy combines the advantages of imaging thin sections of a cell spheroid with the capability to image deep within the spheroid.

**Fig 3:**
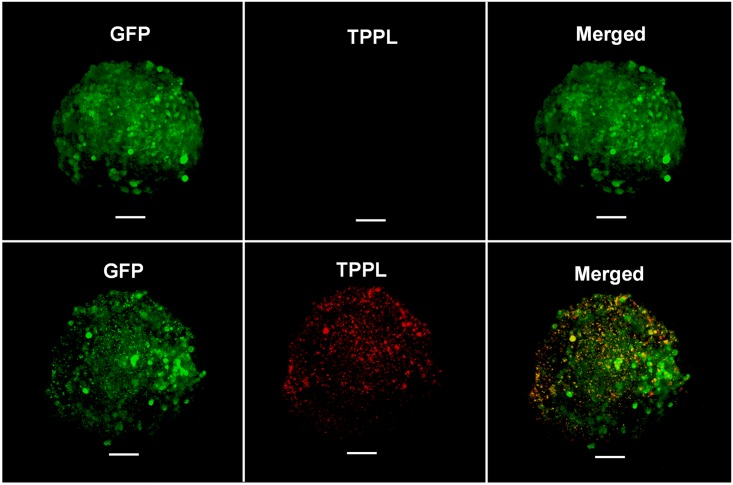
Sample Two-photon images of HCT116 cell spheroids used for estimation of AuNP uptake in cell spheroids. The images in the top row are obtained from a control spheroid while the images in the bottom row are obtained from a cell spheroid incubated with AuNP. The green emission is due to TurboGFP expressed by the HCT116 cells while the red emission is from the AuNP. (Scale bar: 50μm).

Representative two-photon images of a pair of spheroids are shown in Fig 3. The two-photon emission from the metal nanoparticles is clearly visible in Fig 3. Interestingly, we were unable to reliably detect a gold signal using ICP-MS in the same sample size, demonstrating the superior sensitivity of two-photon microscopy to ICP-MS for sensing of AuNP uptake in cell spheroids.

To quantify the distribution of the nanoparticles within the spheroids, we developed custom software in MatLab. The software sections a single optical slice of a spheroid into a series of elliptical rings as shown in the supplement (Fig D in S1 File). The average AuNP emission intensity in the individual rings is then used as a proxy for AuNP concentration in each ring. Notably, since the AuNP concentration is measured as a function of the distance from the spheroid surface, the measurements are not affected by variations in spheroid size. Additional details are included in the Methods section and the supplementary information (Figs E, F and G in S1 File).

**Fig 4:**
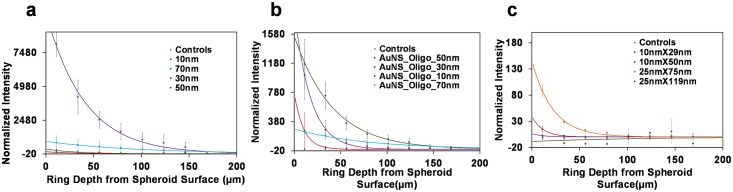
AuNP TPPL intensities measured at various depths from the surface of cell spheroids after 72 hour incubation. a) four different AuNSs, b) four different AuNS-oligos functionalized with 28 base long oligonucleotides and c) four different AuNRs, Note that the controls and low intensity measurements can be slightly negative due to background subtraction (details in Methods).

Fig 4 summarizes the two photon emission intensities for three independent spheroids for each condition as a function of the depth from the spheroid surface. The data included in Fig 4 is collected from spheroids incubated with corresponding particle type for 72 hours. To quantify the penetration depth and AuNP uptake efficiency, the data points in Fig 4 for each AuNP are fitted to an unweighted exponential, y = ae^-bx^ where x is the distance from the spheroid surface. By setting the exponential equal to the mean + 2σ_control intensity_ in the corresponding subfigure of Fig 4, the AuNP penetration depth can be determined for each AuNP. The area below the exponential curve serves as a proxy for the overall particle uptake in a thin section of tissue along a radial line extending from the surface of the spheroid to the center of the spheroid. All values are included in Table 1. It is important to note that all AuNP intensities are normalized to correct for inherent differences in TPPL intensities of individual AuNP, thus, enabling direct comparison between uptake of different AuNP based on observed TPPL intensity.

**Table 1:** A table of exponential fit parameters to AuNP uptake data in Fig 4.

	a (Arbitrary Units AU)^1^	b (#/μm)	Adjusted R^2^	Area below exponential fit curve (0–200μm AU-μm)^2^	Penetration^3^ depth (μm)
**AuNS**					
10nm	150.3 (37.74, 262.8)	0.034 (0,0.068)	0.605	4414.4	81.
30nm	10440 (9323, 11550)	0.025 (0.021,0.028)	0.991	421457.7	280
50nm	352.6 (174.3, 530.9)	0.032 (0.010,0.054)	0.827	10919.4	113
70nm	913.4 (805.5, 1021)	0.011 (0.008,0.013)	0.972	76337.9	415
**AuNS-Oligo**					
10nm	777.1 (538, 1016)	0.100 (0.074,0.126)	0.995	7789.6	40
30nm	1921 (1749, 2093)	0.057 (0.051,0.063)	0.997	33713.2	86
50nm	1558 (1440, 1676)	0.024 (0.022,0.027)	0.995	63769.8	195
70nm	278.4 (255, 301.7)	0.013 (0.011,0.015)	0.989	19861.1	228
**AuNR**					
10nmx29nm	39.64 (30.01, 49.28)	0.087 (0.067,0.107)	0.995	458	37
10nmx50nm	6.462 (0.703, 12.22)	0.087 (0.014,0.160)	0.927	74.2	16
25nmx75nm	146.3 (127, 165.6)	0.046 (0.038,0.054)	0.993	3186.3	99
25nmx119nm	-8.656 (-34.82, 17.5)	0.009 (-0.038,0.057)	-0.094	-801.1	N/A

^1^ Exponential fit equation: y = ae-bx where y is normalized intensity, x is distance from spheroid surface in μm and a, b are constants. The numbers in brackets indicate the 95% confidence bounds for the fit parameters. The fit for 25nmX119nm sample is poor as indicated by either poor adjusted R square value or confidence interval for the parameter b ranging from negative to positive values.

^2^ The area below the exponential fit curve is calculated for the depth range of 0 to 200μm from the spheroid surface.

^3^ Penetration depth is calculated as the distance from the spheroid surface at which the exponential fit for a particular AuNP sample is equal to the mean of control spheroid TPPL intensity plus two times the standard deviation of the control spheroid TPPL intensity.

As expected, an exponential function generates very good data fits for most of the AuNPs that we tested, as indicated by the adjusted R-squared values in Table 1. The exception was the 25nmX119nm AuNR. The data for this particle was extremely close to the noise, indicating no detectable uptake of these particles and preventing the generation of a sensible data fit.

Interesting trends emerge from the data shown here. The 30nm diameter nanospheres clearly had the highest uptake efficiency, in terms of total AuNP quantity per spheroid as indicated by the plot in Fig 4a and the area below the exponential fit curve in Table 1. Surprisingly, the 30nm and 70nm diameter nanospheres were able to reach large depths within the spheroid, although the mass concentration of the 70nm particles was significantly lower. In comparison, uptake of 10nm and 50nm particles, however, was markedly decreased, and the penetration depth of these particles was less than half of the depth of the 30nm and 70nm nanospheres.

Surface functionalization has previously been used as a way to tune the uptake efficiency of nanomaterials. When the AuNS-oligo is compared to the AuNS, the uptake of 50nm and 10nm diameter particles is significantly enhanced (Fig 4b), as expected. The uptake of 30nm and 70nm diameter particles, however, is reduced compared to the non-functionalized particles (Fig 4b, Table 1). Interestingly, the nearly flat concentration gradient of the 70nm particle in the spheroid body is also observed for both bare and oligonucleotide functionalized particles. The relatively gentle reduction of the concentration of the 70nm particles in the spheroid body is further corroborated by the exponential fit parameter ‘b’ (a concentration decay rate) for these particles being the smallest of all other particles (Table 1)

The results from the studies with rod-shaped particles (AuNR) are included in Fig 4c. When compared to the AuNS results, the uptake efficiency is decreased in general for all AuNR aspect ratios and sizes we tested. Furthermore, the uptake of the AuNR with the highest efficiency appears to be limited to approximately 100μm from the spheroid surface. For comparison, the AuNS with most efficient uptake traveled more than twice the distance into the spheroid structure. The uptake efficiency of AuNR with lower aspect ratios (3:1) was found to be higher for both AuNR diameters (10nm and 25nm) tested than that for AuNR with higher aspect ratio (5:1). The intensity signals from the 25nm diameter higher aspect ratio AuNR were essentially noise, indicating minimal uptake into the spheroid.

Corresponding data obtained from spheroids incubated with all different types of AuNP for 24 hours is included in the supplement (Fig H in S1 File). This data indicates that the uptake efficiency of all bare particles (spherical and rod shaped) was extremely low at this time point as compared to the data shown in Fig 4, indicating a slow uptake of bare particles in the spheroid body. In contrast, the oligonucleotide functionalized particles were present at comparable levels in spheroids at the 24 hour incubation timepoint as compared to the 72 hour incubation data (Fig 4b), indicating relatively rapid uptake kinetics of these particles.

**Fig 5:**
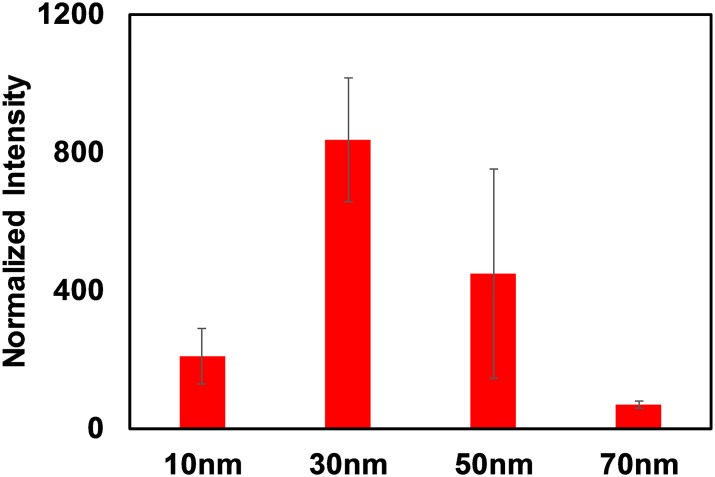
AuNP TPPL intensities measured from 2D monolayer samples of HCT116 cells incubated with different sized spherical AuNP.

For a direct comparison with the same cell type in 2D, we incubated a 2D monolayer of HCT116 cells with the AuNS. The average normalized AuNP intensities obtained through two-photon microscopy are plotted in Fig 5. If the data from the 2D and 3D measurements is compared, the particle with the best uptake is consistently the 30nm diameter. The uptake levels of the other particle sizes, however, differ considerably across the two cell culture types, demonstrating that the 2D culture is not able to accurately predict the entire behavior of the 3D system. Most likely, the main contributor is the lack of the inter-cellular nano-architecture in the 2D culture. This architecture can play a significant role in the diffusion and transport of AuNPs. It should be noted that the normalized intensities plotted in Figs 4 and 5 are a proxy for the mass concentration of the respective particles (i.e. gold content per unit volume of the spheroid) in contrast to the gold nanoparticle count per cell included in a number of publications characterizing gold nanoparticle uptake in cells[13,21,22].

We attempted a similar comparison for the AuNR. However, the HCT116 cells cultured in 2D were unable to survive the incubation with the AuNR at identical concentrations to those used in the 3D spheroids (Fig C in S1 File). At lower concentrations, we were able to achieve high cell survival. However, TPPL intensity obtained from any AuNR taken up by the cells was too weak for detection above the background levels.

## Conclusions

In conclusion, by leveraging the intrinsic two-photon emission of AuNP in combination with two-photon photoluminescence microscopy, we were able to determine the distribution of AuNS, AuNS-oligos, and AuNR in 2D and 3D cultures of live cells. Upon exposure to the AuNPs, the two cell culture methods exhibited markedly different cell growth and uptake efficiencies, providing further evidence that experimental results obtained from 2D cultures are not indicative of the 3D behavior. Additionally, this study revealed that smaller spherical nanoparticles are able to more efficiently penetrate into the spheroid than rod-shaped nanoparticles. This finding is of critical importance as researchers are actively developing cancer therapies based on gold nanorods, and tumor penetration depth is directly tied to therapeutic efficacy. More generally, the use of two-photon photoluminescence microscopy to characterize nanoparticle uptake in cell spheroids and tissue to optimize therapeutic efficacy will find numerous applications beyond the specific nanoparticle geometries and cancer line studied here.

## Methods

### 2D and 3D cell spheroid culture

TurboGFP expressing HCT116 cells, a colorectal carcinoma cell line, was obtained from our collaborators at University of Southern California (USC). The HCT116 cell line was originally obtained by our collaborators from ATCC and then transduced with MISSION TurboGFP^™^ Control Transduction Particles (SHC003V, Sigma-Aldrich Co., LLC). This cell line was maintained in 2D culture in McCoy’s 5A (Modified) medium (Thermo Fisher Scientific, Inc, Catalog: 16600–082) containing 10% fetal bovine serum (Thermo Fisher Scientific, Inc, Catalog: 10082139), 2% Penicillin-Streptomycin (Thermo Fisher Scientific, Inc, catalog: 15140122). Cells were cultured to form 3D cell spheroids using hanging drop 96 well plates according to manufacturer’s instructions (Perfecta3D, 3D Biomatrix, Inc). Briefly, cells were released from 2D culture plates using Trypsin-EDTA treatment and diluted in fresh McCoy’s medium. Cells were counted (Countess II, Thermo Fisher Scientific, Inc), and the cell suspension was diluted to a concentration of 2500 cells/mL. A 40uL volume of the cell suspension is then seeded per well into the hanging drop plates (approx. 100 cells/well). Cell spheroids were allowed to grow for four days, prior to the addition of AuNP to the growth medium. Medium was exchanged according to manufacturer’s instructions by removing 14uL of old media from each well and adding 20uL of fresh medium to account for medium evaporation on day 2. On day 4, medium exchange was performed with fresh medium for control spheroids and experimental spheroids to be tested for 24 hour incubation with AuNP. In a different set, this exchange was performed with medium containing AuNP for experimental spheroids to be tested for 72 hour incubation with AuNP. Then another medium exchange was performed on day6, with medium containing AuNP, for the wells containing experimental spheroids to be tested for 24 hour incubation with AuNP. The medium containing AuNP was maintained at a constant mass concentration of 0.183μg/mL for all AuNP. All the spheroids were imaged on a two-photon microscope on day 7 of each experiment. Spheroids were also imaged using phase contrast microscopy on days 4, 5, 6 and 7 to monitor the growth rate of the spheroids.

For the experimental results indicated in Fig 5, the cells were seeded with medium containing AuNP at a seeding density of 35,000 cells per 8.8cm^2^ cell culture plate. Cells were allowed to grow for three days in medium containing AuNP at a constant mass concentration of 3.5μg/mL. Note that this concentration is higher than that used for the experiments with spheroids since the emission from spherical AuNP taken up by cells in a 2D monolayer at the concentration used for spheroids was found to be too weak for quantification. The experimental result shown in Fig C in S1 File is obtained using AuNR at a concentration of 0.183μg/mL, identical to that used for experiments with spheroids. Before imaging, cells were washed with sterile PBS to remove residual AuNPs not taken up by cells. The cells were then submerged in McCoy’s 5A medium free of phenol red (VWR Corp, Catalog: 95053–172) for two-photon imaging.

### AuNP functionalization and characterization

Spherical and rod shaped AuNP were purchased from Nanopartz, Inc. The AuNS consisted of 10nm (SPR peak: 514-520nm), 30nm (SPR peak: 523nm), 50nm (SPR peak: 531nm) and 70nm (SPR peak: 542nm) diameters, while the AuNR consisted of 10nmX29nm (SPR peak: 700nm), 10nmX50nm (SPR peak: 900nm), 25nmX75nm (SPR peak: 700nm) and 25nmX119nm (SPR peak: 980nm). The integrity of the AuNP samples was tested through UV-Vis spectrophotometry (DU 730, Beckman Coulter, Inc.) as shown in the supplement prior to experiments.

For the experiments with the AuNS-oligos, the spherical nanoparticles were functionalized with thiolated oligonucleotides using a protocol based on a previously published article[14]. The 28 base long oligonucleotide used for AuNS functionalization (Seq: 5’- DTPA-TT TTT TTT TTT TTT TTT TTT TTT TTT TT—Alexa594—3’) was synthesized by Integrated DNA Technologies, Inc. The conjugation reaction mixture consisted of 25ug/mL AuNS, 1X sterile PBS, 0.01% SDS and 0.3M NaCl (final concentration gradually increased in three steps). The reaction mixture was incubated on a shaker for 24 hours. After incubation, AuNS samples were centrifuged and washed with 1X PBS to remove unreacted oligonucleotides and other reagents from the AuNP solution. The functionalization of the AuNS with oligonucleotides was then verified using fluorescence spectroscopy (details in supplementary information). The oligo-conjugated AuNS were stored at 4°C prior to incubation with cell spheroids.

### 3D spheroid growth rate calculation

Images of spheroids were taken on days 4, 5, 6 and 7 using phase contrast microscopy. The outer boundary of each spheroid in each image is marked by an ellipse manually using a simple GUI in Matlab. This ellipse then provides X and Y semi-axial lengths of the ellipsoid approximating the shape of the spheroid. The Z semi-axial length of the ellipsoid is approximated as the average of the X and Y semi-axial lengths. The volume of each spheroid is then calculated as the volume of the ellipsoid with these three semi-axial lengths. These volume calculations are then used to calculate the growth rates shown in Fig 2. For instance, the day4-day5 growth rate for a spheroid is calculated as the log to the base two of the ratio of the calculated volumes of the spheroid on days 5 and 4. To normalize the growth rates of experimental spheroids with the control spheroids, the ratios of the calculated growth rates of all spheroids with the growth rates of all the control spheroids are obtained. The mean and the standard deviation of these normalized growth rates for each class of spheroids is then plotted in Fig 2. Very irregular shaped spheroids were excluded from this analysis to avoid large error in volume estimation resulting from approximation of the spheroid shape with an ellipsoid.

### Two-photon imaging and data analysis

Two-photon imaging was performed on a Zeiss LSM-780 upright confocal microscope at 800nm excitation wavelength. Detection was performed using two Non-Descanned Detectors (NDD) on the microscope. To differentiate the GFP emission from the cells and the TPPL from the AuNPs, two wavelength bands were isolated using band pass filters; specifically, Green (505-545nm) and Red (618.5–675.5 nm) using FF02-525/40-25 (Semrock, Inc.) and FF01-647/57-25 (Semrock, Inc.), respectively. All of the images were acquired using a Zeiss W Plan-Apochromat 20X/1.0 DIC (UV) Vis-IR objective. For each spheroid, a Z stack spanning 560μm was acquired, collecting one frame for every 20μm distance along the Z axis.

Image analysis was performed in custom Matlab software. Since the HCT116 cells used were TurboGFP expressing cells, the image from the ‘Green’ band above could be used to mark the periphery of a spheroid in each image. To perform image analysis, we selected the centermost three frames (the frames for which the spheroid was broadest in extent) from each spheroid. An outer ellipse marking the outer boundary of a spheroid in a frame was drawn manually using the Matlab software. The software then divided up the region enclosed by the outermost ellipse into progressively smaller elliptical rings 22.5μm in thickness. The image from the ‘Red’ band is then processed using these elliptical rings as markers for different depths from the spheroid surface. Initially, the image is corrected by subtracting the average background intensity estimated from four corners of the image outside the spheroid region. Due to the presence of TurboGFP in cells and the variability in TurboGFP expression across the spheroid, a portion of the TurboGFP emission can bleed into the ‘Red’ channel, resulting in false positive emission which can be misconstrued as emission from AuNP. To correct for this, an empirical relationship was established between average TurboGFP intensity from the ‘Green band’ and the average intensity observed in the ‘Red’ band for individual elliptical rings using the control spheroids which were not exposed to any AuNP (Fig E in S1 File). Using this empirical relationship and the TurboGFP intensity for each elliptical ring from an experimental spheroid, we corrected the average AuNP intensity obtained for that ring for additional emission due to bleeding of TurboGFP emission in the ‘Red’ channel. A similar empirical relationship was obtained from the experiments with 2D layers of cells, albeit using average TurboGFP and AuNP intensities observed within the regions containing cells (Fig F in S1 File).

Each individual AuNP can exhibit a slightly different TPPL emission intensity for the same mass concentration and same imaging conditions, thus, making direct comparisons between different AuNPs difficult. To correct for this difference in inherent AuNP emission intensity and to enable direct comparison between AuNP, we obtained emission intensity data for all AuNPs used to obtain experimental results presented in this manuscript (Fig G in S1 File). The intensity data for all the AuNPs, except the 10nm, 30nm, and 50nm AuNS, was obtained at a mass concentration of 100μg/mL. The average emission intensity observed in the ‘Red’ band from the control sample (labeled ‘Fluorescein’ in Fig G top panel in S1 File) was subtracted from the average emission intensity of all AuNP. A ratio of the background corrected emission intensity for each AuNP to that of the emission intensity from the 25nmX75nm AuNP was obtained as a normalizing factor. Since the emission intensity of the 30nm and 50nm spherical AuNP was found to be very weak and close to background levels at a concentration of 100μg/mL, the emission intensity of all spherical AuNP was obtained at a higher concentration of 600μg/mL (Fig G bottom panel in S1 File). The known normalization factor for the 70nm particle determined from the data in Fig G top panel in S1 File and the ratios of the emission intensities of the remaining spherical AuNP with that of the 70nm particle determined from Fig G bottom panel in S1 File were then used to calculate the normalization factors for the remaining spherical AuNP. These normalizing factors were then used to scale the mean intensities and standard deviations of the data corresponding to each AuNP as shown in Fig 4 and Fig H in S1 File to facilitate direct comparison between different AuNP types.

The corrected mean AuNP emission intensity data from each elliptical ring for three central frames from three independent spheroids exposed to the same AuNP incubation conditions was then combined to generate each data point shown in Fig 4 and Fig H in S1 File. These data points are then plotted against the depth of the center of the corresponding elliptical ring from the spheroid surface as shown in Fig 4 and Fig H in S1 File.

## Supporting Information

S1 File Supplementary data. Supplementary material including nanoparticle characterization data, experimental data required for imaging data analysis and additional secondary experimental data is included in this file.
(PDF)
