# Supplementary material for: Two-Photon Microscopy Analysis of Gold Nanoparticle Uptake in 3D Cell Spheroids
Source: PLoS One. 2016 Dec 9;11(12):e0167548. doi: 10.1371/journal.pone.0167548 (PMC5147918; doi:10.1371/journal.pone.0167548)
Supplement: S1 File — Supplementary material including nanoparticle characterization data, experimental data required for imaging data analysis and additional secondary experimental data is included in this file. (PDF) [file pone.0167548.s001.pdf]

*Supplementary Information*

# Two-photon microscopy analysis of gold nanoparticle uptake in 3D cell spheroids

Tushar D. Rane, Andrea M. Armani

## 1. Material Characterization

Initially we verified the integrity of non-functionalized AuNS and AuNR using UV-Vis spectrophotometry. The measured absorption peaks for AuNS and AuNR closely matched the manufacturer's specifications (Fig. A). The absorption spectra shown in Fig. A were obtained at a concentration of 35µg/mL for all AuNP and then normalized to the peak absorption for each AuNP.

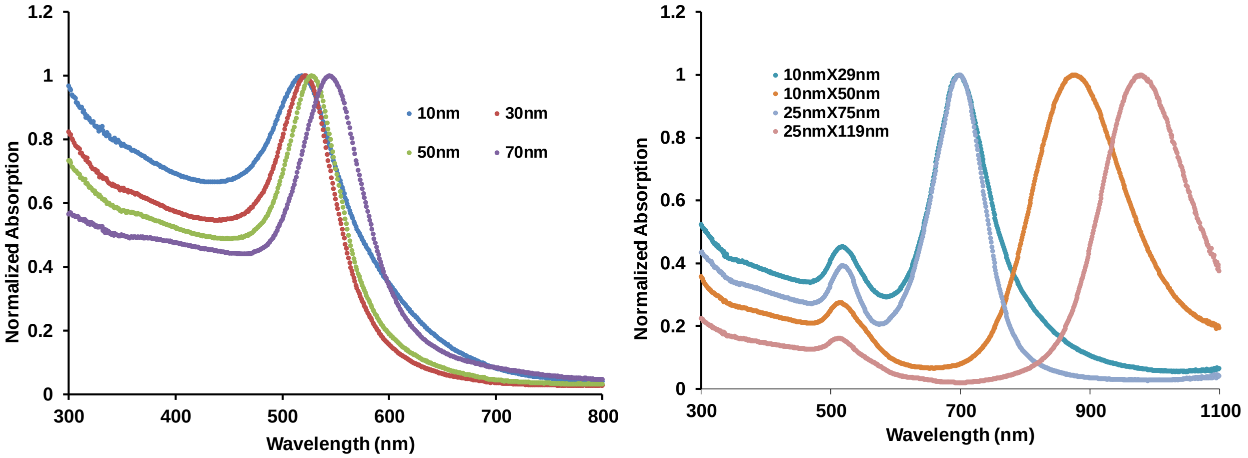


**Fig. A:** UV-Vis absorption spectra of various AuNP used for downstream experiments. Left panel shows normalized absorption spectra for spherical AuNP with diameters 10nm, 30nm, 50nm and 70nm while the right panel shows normalized absorption spectra for rod shaped AuNP with sizes 10nmX29nm, 10nmX50nm, 25nmX75nm and 25nmX119nm. Measured absorption peaks for different particles are as follows: Spherical particles (10nm: 518nm, 30nm: 521nm, 50nm: 527nm, 70nm: 544nm), Rod particles (10nmX29nm: 699nm, 10nmX50nm: 877nm, 25nmX75nm: 700nm and 25nmX119nm: 979nm)

We verified the functionalization of the AuNS with oligonucleotides using fluorescence spectroscopy since the oligonucleotides were labeled the fluorescent dye Alexa594. The fluorescence spectra were collected using excitation at 590nm on a Horiba Fluoromax4 Spectrofluorometer. The emission of fluorescence from all the functionalized AuNS in Fig. B indicates successful functionalization of the AuNS with oligonucleotides.

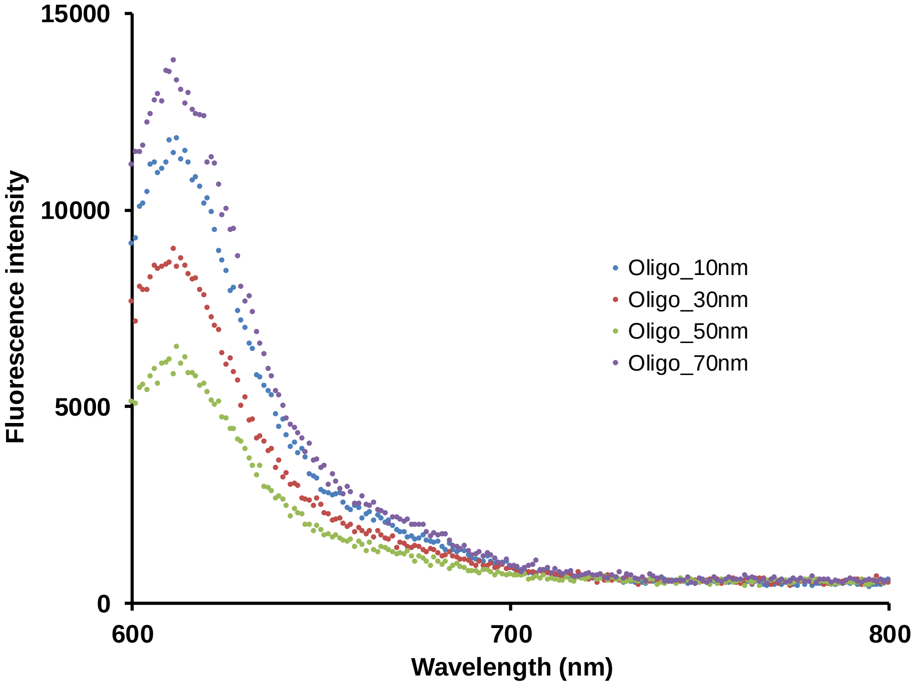


**Fig. B:** Verification of AuNP functionalization with oligonucleotides labeled with Alexa594

## 2. Control Measurements

Fig. C shows sample phase contrast images from monolayers of HCT116 cells exposed to no AuNP (Control), 10nmX29nm AuNR (0.183µg/mL), 25nmX75nm AuNR (0.183ug/mL), 10nm AuNS (3.5µg/mL), and 50nm AuNS (3.5µg/mL) for three days. The round and detached cells exposed to AuNR samples indicate poor survival of these cells in the presence of AuNR at the indicated concentration.

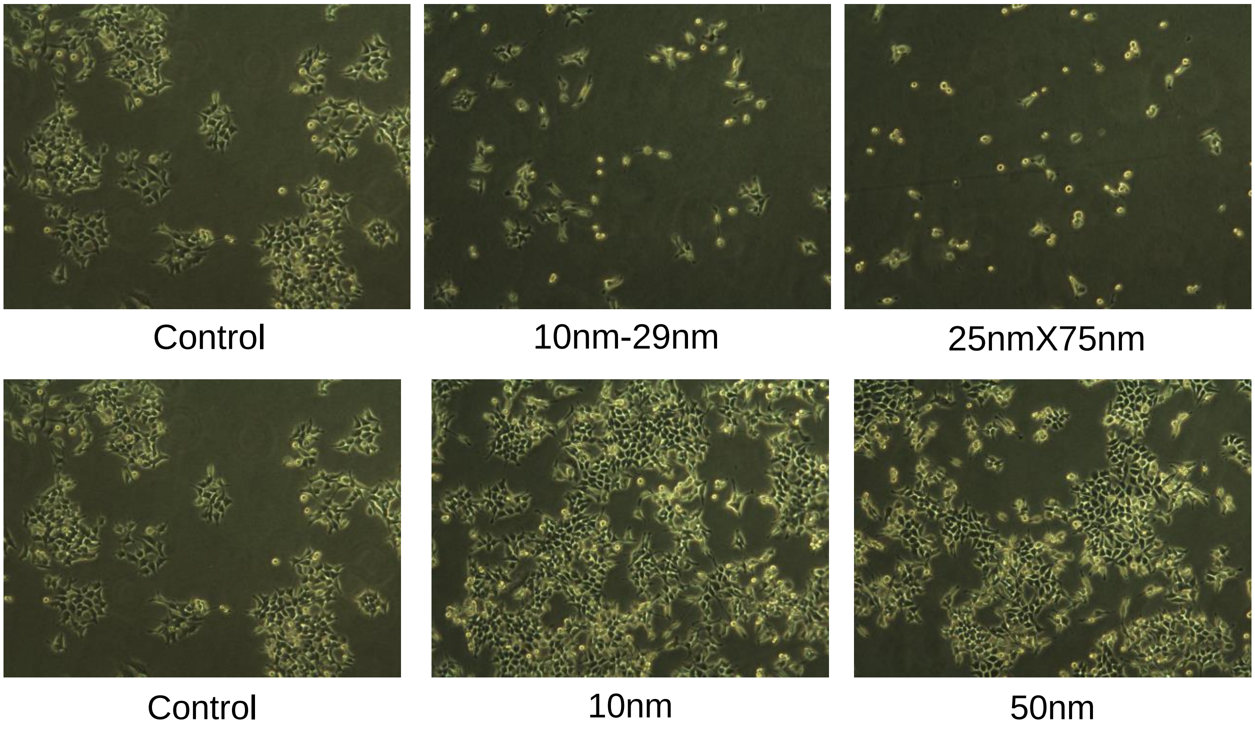


**Fig. C:** Cell death following exposure to AuNR

### 3. Image Analysis and Signal Quantification

Fig. D shows a sample frame from a Z stack of frames obtained from a spheroid using two-photon microscopy. The spheroid cross section in this frame has been divided into progressively smaller elliptical rings with a constant ring thickness of 22.5µm. This constant ring thickness and format of spheroid sectioning was used to analyze data from all spheroids.

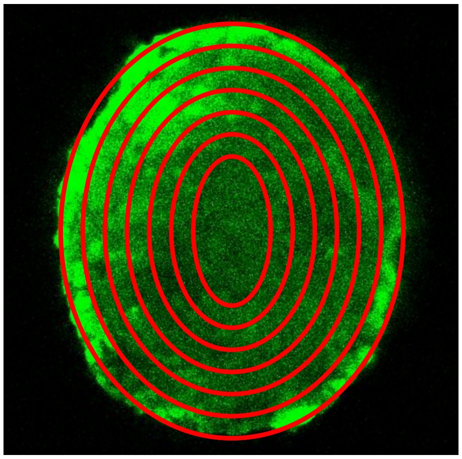

**Fig. D:** Sectioning of a spheroid into progressively smaller elliptical rings

Fig. E and F indicate the empirical relationship obtained between the mean GFP intensity and the corresponding AuNP background intensity observed in control spheroids and control monolayer of cells respectively. These data indicate the extent of bleeding of the emission of TurboGFP from the HCT116 cells in the AuNP channel. The data fits in these figures were used to correct the measured AuNP intensity from spheroids and monolayers of cells for contributions from bleeding over of the TurboGFP emission into the AuNP channel as detailed in the 'Methods' section.

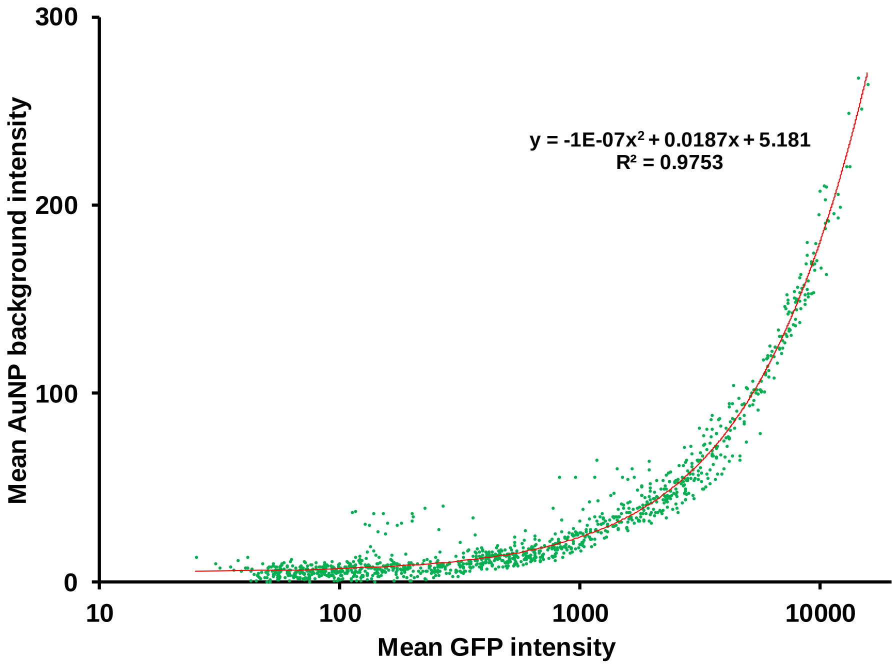


**Fig. E:** Empirical relationship between GFP fluorescence emission intensity and background

intensity measured from the AuNP channel for cell spheroid data. Each data point in the plot indicates average TurboGFP intensity and corresponding average background intensity observed in the Red band corresponding to AuNP from an elliptical ring (as shown in Fig. D) in a control spheroid.

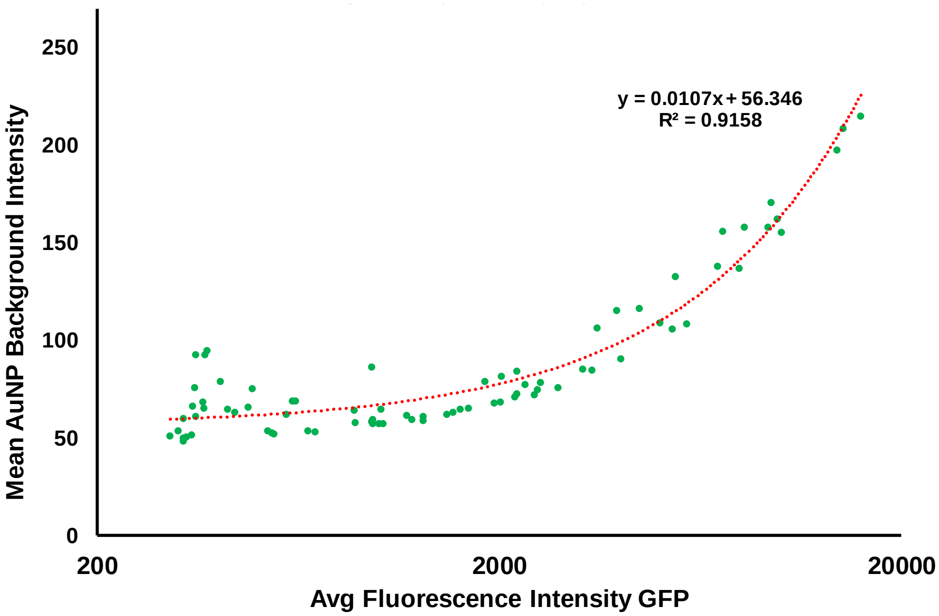


**Fig. F:** Empirical relationship between GFP fluorescence emission intensity and background intensity measured from the AuNP channel for 2D monolayer data. Each data point in the plot indicates average TurboGFP intensity and corresponding average background intensity observed in the Red band corresponding to AuNP from the region containing cells in an image of a 2D culture of control cells.

Fig. G indicates the measured TPPL intensities of different AuNP used in the experiments reported. The TPPL intensities in the top panel were obtained at a fixed concentration of 100 µg/mL for all particles while the TPPL intensities in the bottom panel were obtained at a fixed concentration of 600µg/mL as detailed in the 'Methods' section. These measured inherent differences in TPPL intensities of AuNP were used to normalize the AuNP TPPL intensities measured from spheroids and monolayers of cells to enable direct comparison between different AuNP as detailed in the 'Methods' section.

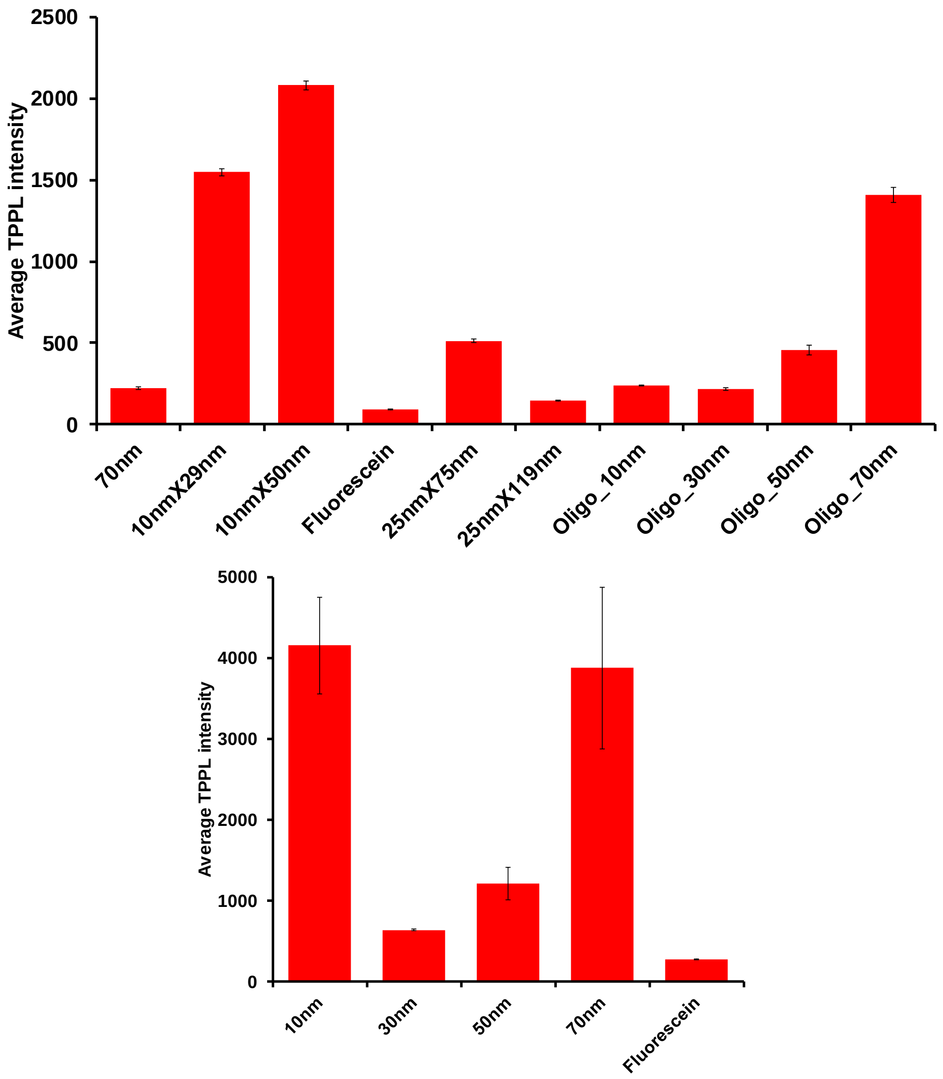


**Fig. G:** AuNP TPPL intensity comparison. Note that the particles in two panels are measured at different concentrations and imaging conditions. The common particle (70nm diameter) is used to compare the TPPL intensities of all particles with each other.

## 4. Additional data

Fig. H shows additional data obtained from spheroids exposed to different AuNP for 24 hours to supplement the data shown in Fig. 4 in the main text, which is obtained after exposure of the spheroids to AuNP for 72 hours.

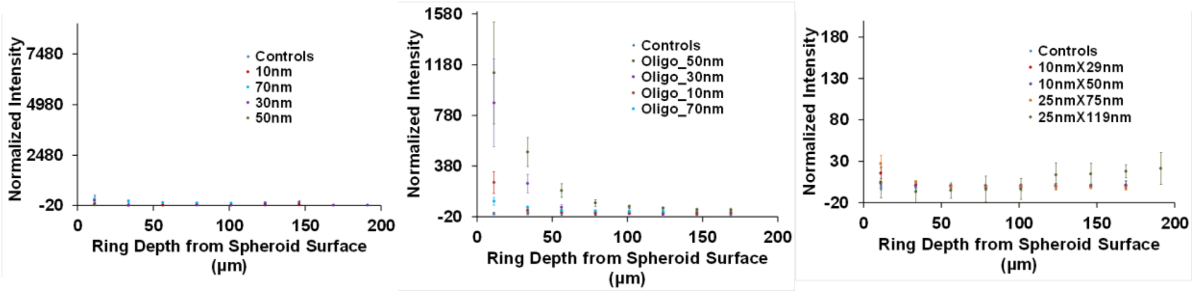


**Fig. H:** AuNP TPPL intensities measured at various depths from the surface of cell spheroids after 24 hour incubation for a) four different spherical AuNPs, b) four different spherical AuNPs functionalized with 28 base long oligonucleotides and c) four different rod shaped AuNPs, Note that the controls and low intensity measurements can be slightly negative due to background subtraction (details in Experimental Methods).
